# One-Stage Exchange Arthroplasty for Fistulizing Periprosthetic Joint Infection of the Hip: An Effective Strategy

**DOI:** 10.3389/fmed.2020.540929

**Published:** 2020-10-16

**Authors:** Simon Marmor, Younes Kerroumi, Vanina Meyssonnier, Luc Lhotellier, Antoine Mouton, Wilfrid Graff, Valérie Zeller

**Affiliations:** ^1^Department of Orthopedic Surgery, Diaconesses Croix Saint-Simon Hospital, Paris, France; ^2^Referral Center for Osteoarticular Infections, Diaconesses Croix Saint-Simon Hospital, Paris, France

**Keywords:** hip, infection, joint, prostheses, one stage exchange arthroplasty, fistula

## Abstract

**Background:** Prosthetic hip infection (PHI) is a disastrous scenario after an arthroplasty. International guidelines contraindicate one-stage exchange arthroplasty for fistulizing chronic prosthetic hip infection (FCPHI), nevertheless few surgical teams, mostly from Europe, support one stage procedure for this indication.

**Questions/Purposes:** Analysis of infection recurrence and implant failure of a series of FCPHIs treated with one stage arthroplasty.

**Patients and Methods:** Sixty-six FCPHIs treated with one-stage exchange arthroplasty were prospectively followed up at least 2 years. Clinical, radiological and bacteriological signs suggestive of reinfection were sought, as well as implant failures and PHI related deaths.

**Results:** Thirty-four females and thirty-two males with median age of 69.5 years [61–77] and BMI of 26 kg/m^2^ [22-31] were included. Fistulae were productive in 50 patients (76%). *Staphylococcus* was responsible for 45% of PHI and 21% were polymicrobial. Twenty-nine patients (44%) received preoperative antibiotic therapy. After a median 60-month follow-up [35–82], 3 patients (4.5%) presented reinfection (two new infections, one relapse) and 3 patients experienced implant failure (1 femoral fracture, 1 stem breakage, 1 recurrent dislocation). One death was related to PHI. After a minimum of 2 years, the infection control rate was of 95.3% (±0.02).

**Conclusion:** One-stage exchange arthroplasty for FCPHIs showed a good infection control rate similar to that of non-fistulizing PHI. Systematic preoperative microbiological documentation with joint aspiration and, in some specific cases, the use of preoperative antibiotic therapy are among the optimizations accounting for the success of the one-stage arthroplasty. In light of these results, and those of other studies, international recommendations could evolve.

**Level of Evidence:** Descriptive therapeutic prospective cohort study. Level of evidence: IV.

## Background

The treatment of chronic periprosthetic hip infection (PHI) is still a controversial issue. There are currently two conventional surgical treatment procedures. The two-stage exchange arthroplasty is the most common treatment worldwide; nevertheless, a one-stage exchange procedure is gaining more and more ground (1–4). This technique is encouraged by satisfactory results of infection control rate in selected patients, at a minimum follow-up of 2 years [Wroblewski et al. (5), 91%; Loty et al. (6), 91%; Raut et al. (7), 86%; Winkler et al. (8), 92%; Klouche et al. (9), 100%; Hansen et al. (10), 70%; Choi et al. (11), 82%; Zeller et al. (12), 96%].

Other obvious benefits of one-stage surgery are the reduction in cost-burden, operating time, anesthetic risk, and complications inherent in multiple hospitalizations and surgeries.

The choice between those two strategies is guided by bacteria nature and its antibiotic susceptibility, PHI prior treatment, bone quality, patient's underlying conditions, and soft tissue inflammatory state, which indicates when severe, two-stage arthroplasty according to some authors (13–18).

Studies on fistulizing chronic periprosthetic hip infections (FCPHIs), treated with one-stage arthroplasty, are scarce and report only a few cases of FCPHIs with satisfactory infection control (5, 12, 19, 20). To our knowledge, only one prospective study described specifically the results of a series of 57 PHIs with productive fistulae, reporting a rate of 86% of reinfection-free survival after a mean follow-up of 7 years (7). Although no studies have compared one- and two-stage arthroplasty in FCPHI treatment, expert panels and international recommendations favor the two-stage strategy, arguing the likelihood of an assumed higher risk of reinfection with one-stage surgery in this indication (15, 17, 21–23).

Therefore, we asked (1) what is the reinfection-free survival rate after one-stage arthroplasty revision for patients with FCPHIs at a 2-year follow-up? (2) What is the implant failure-free survivorship for the same patients at the same follow-up?

## Materials

### Study Population

Patients included were sampled from a cohort of 541 PHIs between 2003 and 2014. Three hundred and seventy-three were managed with one-stage exchange arthroplasty, 97 with two-stage surgery, 30 with debridement, antibiotics, and implant retention, and 41 with other strategies (resection, delayed reimplantation). In this cohort, the presence of a fistula was never a contraindication to performing one-stage arthroplasty. Until 2008, two-stage strategy indications were either major bone defects or unknown PHI-causative germ. Afterward, we performed a one-stage exchange arthroplasty to almost all PHIs.

We included in this single-center, prospective cohort study patients over 18 years of age undergoing one-stage exchange arthroplasty for FCPHI in our referral center of osteoarticular infection.

### Endpoints

The primary endpoint of the study was the occurrence of prosthetic hip reinfection. Reinfection corresponds to a recurrence of the prostheses infection, which could be either a relapse with the same bacteria or a new prosthetic infection due to a different one. The secondary endpoint is the occurrence of implant failure. It may be a loosening, dislocation, or any other mechanical event occurring in the patient's prostheses, without any clinical, biological, or radiological sign suggesting a PHI. In addition, the cultures of preoperative joint aspiration fluid and intraoperative samples must be sterile in case of revision.

### Methods

All patients were treated and followed at least 2 years after surgery. They were reviewed at the end of the antibiotic therapy period (3 months) and then, at 1 year, 2 years post-operatively, then every 2 years. Phone interviews were conducted to gather the latest news from patients who were unable to attend follow-up visits.

At each visit, we sought clinical (pain, fever, local inflammation), radiological (appearance of periosteal bone apposition/radiolucent line, geodes…), and biological [increase in C-reactive protein (CRP)] and polymorphonuclear neutrophil count signs suggestive of reinfection or implant failure. Deaths were monitored as well. In the absence of clinical, biological, and/or radiological signs of infection, PHI was considered healed after 2 years of follow-up (24).

### Ethics Statement

All participants were informed and gave their consent before the start of this study, which was approved by the Local Ethics Committee.

### Statistics

Qualitative variables were described according to frequency. Quantitative variables were assessed for normality. They were described by their mean and standard deviation (Sd) if they met a normal distribution, otherwise by their median and interquartile range. They were compared from baseline to 24th month using the non-parametric Wilcoxon signed-rank test. The reinfection-free survival of implant failure and PHI-related mortality was analyzed using Kaplan–Meier's method and expressed as a rate with its Sd. Log-rank (Mantel–Cox) test was used to compare the survival distributions of the two groups. A *p* < 0.05 was considered significant. All statistical tests were performed with SPSS.20 software.

### Diagnosis and Therapeutic Strategy

PHI diagnosis was based on the presence of one or more fistulae, which is a major criterion for periprosthetic joint infection diagnosis (24, 25), and confirmed by the results of microbiological cultures of preoperative joint aspiration and/or intraoperative samples. For infection recurrence, the diagnosis was established through the same workup as the initial diagnosis.

The pathogen was considered causative of PHI when it was isolated from ≥2 different intraoperative specimen samples or joint fluid aspirates. The diagnosis and surgical strategy for all patients were validated during the weekly multidisciplinary consultation meeting, involving at least one orthopedic surgeon, one infectiologist, and one microbiologist.

At least 2 weeks after discontinuing any ongoing antibiotic therapy, preoperative aspiration of the joint fluid was done in the Department of Radiology under fluoroscopic guidance and strict sterile conditions. In addition, two joint washing-aspirations with the saline solution were performed. Specimens were intended for the determination of differential white blood cell counts and microbial identification.

Joint aspiration was completed with media contrast injection to view the fistula pathway via arthrography.

One-stage exchange arthroplasty was the surgical technique adopted in this series. It involved the excision of the old scar and the fistula pathway through the former incision or a new one to permit a double approach.

After thorough debridement, the old prosthesis was removed. In some cases, trochanterotomy and/or femorotomy were carried out to facilitate the endofemoral cement excision, implant extraction, and joint exposure.

Debridement consisted of an extensive and circumferential synovectomy. All macroscopically infected or suspect tissues were excised. Osteosynthesis hardware and cement were removed. During the surgical excision procedure, at least five intraoperative specimens were sampled from synovial, acetabular, and femoral sites. Specimens were immediately transported to the laboratory of microbiology, then diluted and crushed. Afterward, the final suspension was aliquoted and cultured. When necessary, non-antibiotic-impregnated bone allograft was performed to fill the bone loss. Finally, the new prosthesis was implanted after one saline washing. Most of the time, the implant was cementless, and when cemented, no antibiotics were added. All patients had drain suction during 3–5 days post-operatively.

Antibiotics susceptibility testing was performed for all isolated germs, according to the recommendations of the French Society of Microbiology (26).

Polymicrobial infection included different genera. The presence of different staphylococcal species defined mixed staphylococcal PHIs. The antibiotic therapy was initially guided by the results of the culture of the preoperative joint aspirate and subsequently adapted to the microbiological results of the intraoperative samples.

PHI was classified according to Tsukayama's classification (27); two PHI groups were considered post-operatively acquired, i.e., without signs of hematogenous spread. Early-post-operative infection was defined as surgical site pain, redness with or without drainage, associated or not with fever, occurring within 30 days after joint arthroplasty. Late-chronic infection was defined as progressive pain, joint dysfunction with or without a fistula, occurring ≥1 month after joint arthroplasty.

A hematogenous infection was defined as occurring after a symptom-free interval of ≥1-month post-surgery, with sudden onset of pain, joint dysfunction with or without fever, and/or chills, a virulent bacterium compatible with hematogenous dissemination (*Staphylococcus aureus, Streptococcus, Enterobacteriaceae*…), or identification of a portal of entry.

All patients received post-operative antibiotic therapy, which was launched intraoperatively with at least one intravenous (IV) antibiotic through a central venous catheter. Continuous infusions administered vancomycin, cefazolin, ceftazidim, piperacillin-tazobactam, and clindamycin. The monitoring of antibiotic serum levels was performed for all IV antibiotics. Fusidic acid, minocycline, levofloxacin, and linezolid were administered by oral regimen (28–31).

When the result of preoperative joint fluid culture identified monomicrobial infection with *S. aureus, Streptococcus* sp., *Enterobacteria*, or *Pseudomonas aeruginosa*, preoperative antibiotic therapy was initiated.

The duration of post-operative IV antibiotic therapy was 4–6 weeks, relayed by an oral regimen for a total duration of 12 weeks, in accordance with French and international recommendations (21, 32).

At the beginning of this cohort study, all patients received 6 weeks of IV antibiotics and 6 weeks of an oral regimen. From 2008, we decided to decrease the duration of the IV phase to 4 weeks if PHI was due to an organism deemed susceptible, such as methicillin-susceptible *Staphylococcus* and/or anaerobes from the skin flora.

## Results

### Baseline Characteristics

Sixty-six FCPHIs occurred in 66 patients (34 females and 32 males) with a median age of 69.5 years [61–77] and body mass index of 26 kg/m^2^ [22–31]. Osteoarthritis was the indication for the index implantation of a hip prosthesis in 46 cases (70%), followed by fractures in 15 cases (23%). Forty-one patients (62%) had cardiovascular history, 15 (23%) had diabetes, 8 (12%) dyslipidemia, 6 (9%) thromboembolism disorder, 5 (8%) hepatitis, 7 (11%) cancer, 3 (5%) renal failure, and 3 (5%) had inflammatory rheumatism. The American Society of Anesthesiologists (33) score was grade I in 3 (5%) patients, II in 46 (70%), III in 16 (24%), and IV in 1 (1%). Twenty-two patients (33%) experienced prior medical–surgical treatment failure of their PHI in other hospitals (19 debridement, antibiotics, and implant retention, 2 one-stage exchange arthroplasties, and 1 two-stage exchange arthroplasties). Nineteen (29%) underwent prior failed antibiotic therapy without surgery.

### Infection Description

According to Tsukayama classification, the initial infection mechanism was for 16 (24%) PHIs as early post-operative (<1 month), 30 (45%) as late post-operative (>1 month), 12 (18%) as hematogenous, and 8 (12%) as undetermined (27).

At the time of PHI treatment in our department, all patients have a chronic infection with symptoms duration >30 days. The median symptoms duration of this series was 241 days (100–530) before one-stage surgery.

Sixty-one patients (92%) had a single fistula, 4 patients (6%) had two fistulae, and 1 had three. On baseline visit, fistulae were productive in 50 patients (76%).

*Staphylococcus* was the most frequent-isolated bacteria, responsible for 30 (45%) PHIs, of which 15 (23%) were due to methicillin-resistant strains, whereas 14 (21%) PHIs were polymicrobial (Table 1).

**Table 1:** Infecting organisms in their frequency.

**Germes**	**Cases**	**%**
*Staphylococcus*/[MR^*^]	30/[15]	45/[23]
*Staphylococcus aureus*/[MR]	16/[6]	24/[9]
*Staphylococcus epidermidis*/[MR]	11/[8]	17/[12]
*Staphylococcus* CN^**^/[MR]	3/[1]	5/[2]
Polymicrobial	14	21
Mixed staphylococcus species	4	6
Mixed bacteria	10	15
*Streptococcus*	4	6
GNB^***^	5	8
*Escherichia coli*	2	
*Pseudomonas aeruginosa*	1	
*Serratia marcescens*	1	
*Prevotella nigrescens*	1	
*Propionibacterium* sp.	4	6
*Corynebacterium* sp.	3	5
*Enterococcus faecalis*	2	3
Negative culture	1	2
Other^****^	3	5

* *Methicillin-resistant*.

** *Coagulase-negative*.

*** *Gram-negative bacillus*.

**** *Finegoldia magma, mycobacterium tuberculosis, peptostreptococcus micros*.

### Initial Workup

Radiographs showed in 14 patients (21%) both acetabular and femoral loosening, in 11 (17%), an acetabular, and in the other 11 (17%), a femoral loosening. Median CRP was 27 mg/l (11–56), and the median leukocyte count was 7,580/mm^3^ (6,475–8,800).

**Figure 1:**
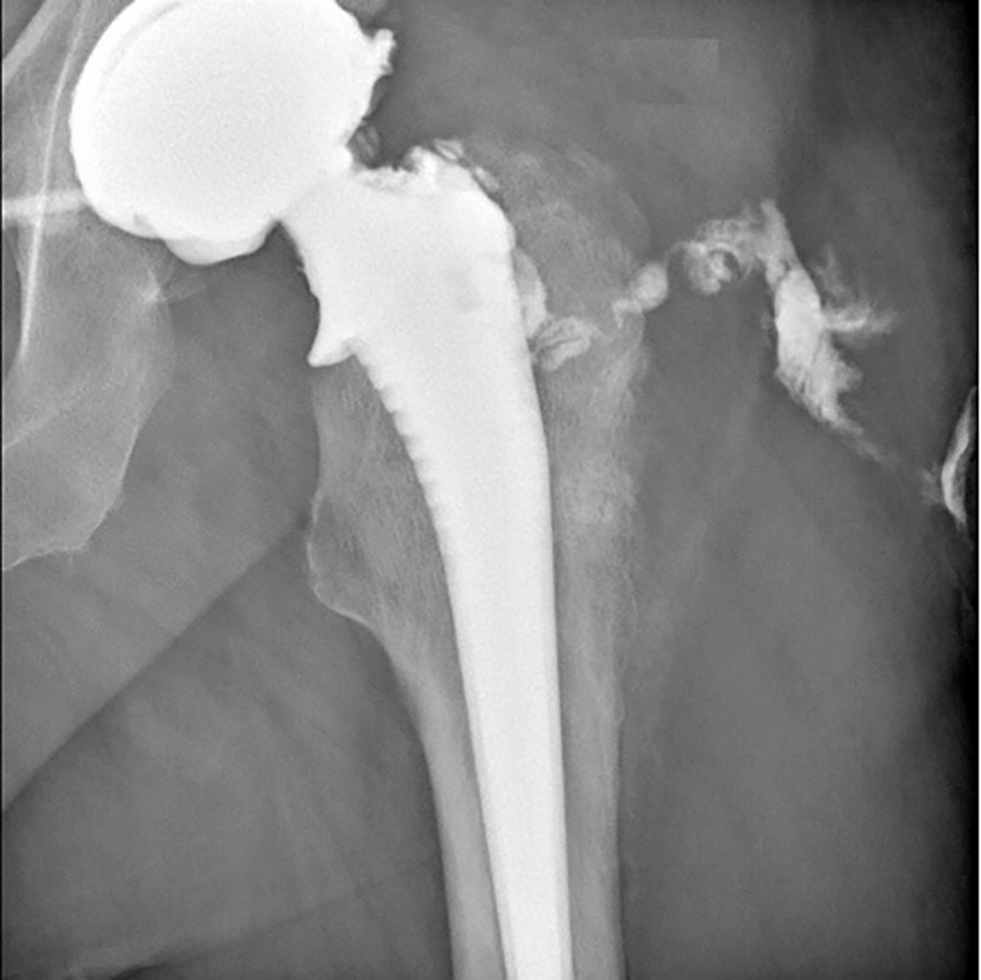
Arthrography showing the fistula pathway.

Preoperative joint aspiration was performed in all patients, arthrography in 54 patients (82%), showing in 41 cases (62%) a communicating pathway between the fistula and joint space (Figure 1). The culture of joint fluid aspirate was positive in 63 cases (95%). It yielded the same bacteria as the intraoperative samples culture in 48 cases (73%). Among the three negative joint fluid cultures, two had positive and one negative intraoperative culture. The latter was operated for an abscess before the exchange arthroplasty. The intraoperative samples yielded *Streptococcus agalactiae*, considered PHI-causative bacteria. Sonication has not been performed for the three negative joint aspiration cultures because our lab was not equipped with a sonication device at that time.

### Antibiotic Therapy

Twenty-nine patients (44%) received preoperative antibiotic therapy with a median duration of 4 days (2–9). In all other cases, antibiotic therapy began intraoperatively after bacteriological samples had been taken from the surgical site.

The median duration of total antibiotic therapy was 84 days (83–90), of which 42 days (30–43) were IV and 42 days (41–55) were oral.

### One Stage Surgery Procedure

One-stage exchange arthroplasty was performed via posterior approach in 50 patients (76%), combined with a double approach to excise a distinct fistula pathway in 13 patients (20%) and via direct anterior approach in 3 patients (5%). A femorotomy or trochanterotomy was necessary in 30 cases (45%). Reimplantations were mostly cementless (45 cases or 68%); the others were cemented without antibiotic-loaded cement. Eleven patients (17%) received an acetabular bone graft to fill bone defects (four graded as Paprosky type 2A, 1 as 2B, 4 as 3A, and 2 as 3B). Among them, three also had a femoral allograft (one graded as Paprosky type 1, 1 as 3A, and 1 as 3B) (34, 35).

### Outcomes

The median follow-up was of 60 months (35–82) with an Sd of 31.3. Sixty-five (98%) patients were seen at 24th month post-operatively, and one was called by phone to collect follow-up data of this visit. No patient was lost to follow up.

The functional score for Postel Merle d'Aubigné (36) rose from 12 (9–15) (95% CI 10.8–13.2) preoperatively to 17 (14–18) (95% CI 14.7–16.3) at 2 years post-operatively with a median difference of 3.5 (1–6) (95% CI 2.5–4.4). The three-item scores showed a significant improvement in pain (*p* < 0.0001), mobility (*p* < 0.0001), and function (*p* < 0.0001).

Three patients (4.5%) had reinfections: We observed one relapse due to methicillin-resistant *Staphylococcus epidermidis* in a patient who initially had a late post-operative PHI. Two patients developed a new infection after their initially classified early post-operative PHIs. Their characteristics, treatments, and vital status are summarized in Table 2. Among the patients who underwent preoperative antibiotic therapy, one patient had PHI relapse. Three implant failures occurred in three patients: one case of stem breakage, which required replacement of the femoral stem, one case of recurrent dislocation (four episodes) treated by femoral stem replacement after three failed reductions, and one case of femoral fracture treated by osteosynthesis.

**Table 2:** Details of the three PHI reinfections.

**Initial germ**	Polymicrobial^**^	Mixed *Staphylococcus* species^***^	MRSE
**One stage surgery with femorotomy**	Yes	No	Yes
**Bone graft**	No	No	No
**Reinfection type**	New infection	New infection	Relapse
**Germ of reinfection**	Polymicrobial^*^	*Enterococcus faecalis*	MRSE
**Age (years)**	77	79	65
**Medical history**	Prostate and colon cancer, HBP, pulmonary embolism	HBP, AF under anticoagulant, depression	Diabetes, HBP, gout, systemic scleroderma
**BMI (cm/kg^2^)**	31	38	31
**ASA**	2	3	2
**Number of previous procedures**	1	0	0
**Delay for reinfection (months)**	1	10	21
**Reinfection treatment**	2 stage	PSAT	1 stage
**Vital status**	PHI-unrelated death	PHI-unrelated death	Alive

* *Methicillin-resistant Staphylococcus epidermidis, Klebsiella pneumonia, and Staphylococcus kloosi*.

** *Methicillin-resistant Staphylococcus aureus and Enterococcus faecalis*.

*** *Methicillin-sensitive Staphylococcus aureus and methicillin Staphylococcus epidermidis*.

*MRSE, Methicillin-resistant Staphylococcus epidermidis; PHI, Prosthetic hip infection; HBP, High Blood Pressure; AF, Atrial fibrillation; PSAT, prolonged suppressive antibiotic therapy*.

Nineteen patients died during the observation period, including three females within 2 years after surgery: a 72-year-old patient with several comorbidities (high blood pressure, dyslipidemia, pulmonary embolism, superficial venous insufficiency, chronic ethylism, peripheral arterial obstructive disease, and smoking) died a month and a half after the operation after a lung cancer diagnosed during the preoperative assessment of her PHI. A 77-year-old patient with no medical history died 5 months post-operatively from a pulmonary embolism. The third death, the only one considered related to PHI, occurred at 8 months post-operatively in a 79-year-old patient with numerous comorbidities (cardiac insufficiency, peripheral arterial obstructive disease, high blood pressure, atrial fibrillation, diabetes, renal failure, and progressive cancer). She had post-operative multiple organ failure and died from sepsis due to *Escherichia coli*, not similar to her PHI-causative germ.

The other deaths occurred after 2 years post-operatively and were unrelated to prosthetic infection. Two event-free PHIs in patients who passed away because of a PHI-unrelated cause, <2 years after surgery, were excluded from survivorship analysis.

**Figure 2:**
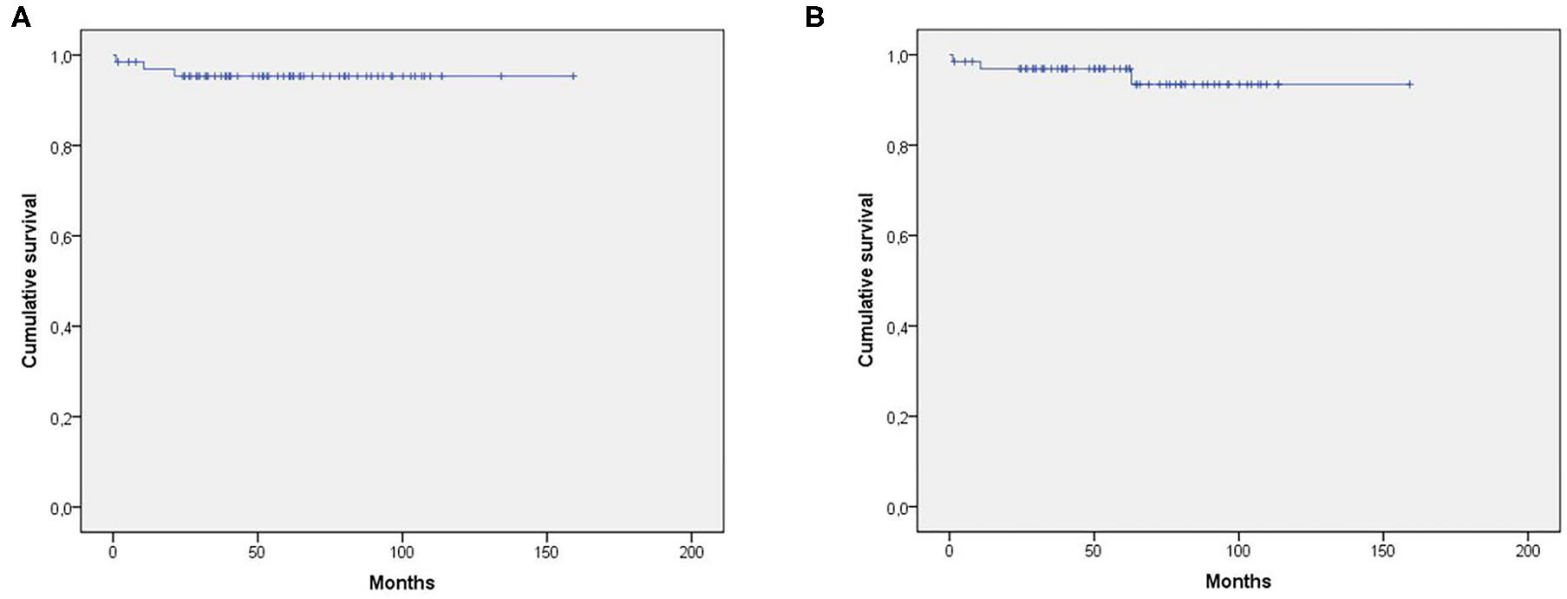
**(A)** Cumulative survival free of reinfection. **(B)** Cumulative implant failure-free survival.

The survival analysis, according to Kaplan–Meier, showed a cumulative reinfection-free survival rate of 95.3% (±0.02) (Figure 2A) and implant failure-free rate of 96.9% (±0.02) at 2 years (Figure 2B). The PHI-related mortality rate was 1.6% (±0.01) throughout the follow-up. The log-rank test showed cumulative reinfection-free survival rates of 100% for patients who had polymicrobial PHI and 94% (±0.03) for those with monomicrobial one (*p* = 0.347).

## Discussion

Since 2003, Zimmerli and his team have consistently proposed two-stage exchange arthroplasty as the surgical treatment of choice for FCPHIs (13, 37). The American recommendations also indicate the same strategy and contraindicate a one-stage procedure in the treatment of prosthetic hip infection with fistula (21, 22).

This choice is justified on the one hand by a risk (deemed high) of failure, due to the mediocre quality of the soft tissues, raising the risk of wound healing complications and, on the other hand, the risk of contamination of the preoperative samples through the fistula, which can prevent the identification of the PHI-causative bacteria.

We reported a series of 66 chronic FCPHI cases treated with one-stage exchange arthroplasty with very satisfactory outcomes. We observed one related death, one relapse, and two new infections, which correspond to a cumulative recurrence rate of 4.7% (±0.02). This rate is not higher than that observed in the literature, in patients treated with one-stage arthroplasty for PHI, without fistula (38).

The outcomes of our study are good and of the same order as those reported by Raut et al. (7), the exclusive series of FCPHIs published in 1994 with an infection control rate of 86%. Other studies in the literature reported series of PHIs with a small proportion of FCPHIs treated with one-stage arthroplasty, achieving success rates comparable to ours [Wrobleski (5), 92%; Hope et al. (19), 85.7%; and Rudelli et al. (20), 93%]. These data are supported by the outcomes of a systematic review of 44 studies, which compared the risk of reinfection between the two revision strategies using pooled individual participant data. Statistical analysis showed that one-stage arthroplasty might be as effective as two-stage in treating PHIs. Surprisingly, the one-stage group had higher CRP levels and a higher proportion of patients with abscess, sinus, draining wound, or fistula, a clinical presentation that often favors the 2-step surgery (39). The authors underlined that the one-stage strategy is an appropriate treatment for a patient with characteristics that had previously been thought to be inappropriate for one stage, such as those with sinus tracts. In addition, a recent study showed that two-stage prosthesis exchange arthroplasty only enables 80% of patients to be reimplanted at the second step (40).

One of the characteristics of our series is the high frequency of polymicrobism, observed in 14 cases (21%), which is higher than in Raut's series (7%) (7), but the same as in Rudelli's one (22%) (20). The presence of a fistula, with a pathway communicating between the joint and the external environment, could lead to superinfection through the fistula of an initially monomicrobial infection. The other reason could be the important frequency of the initially classified acute post-operative PHIs in which polymicrobial PHIs are frequently observed (41).

In this series, no fistula fluid samples were taken into account because we believe that the commensal flora of the skin is likely to be sampled and could skew a microbiological interpretation. For that reason, only joint aspirate was performed preoperatively as well as numerous intraoperative samples to distinguish contaminating from infecting germs.

Kaplan–Meier analysis did not show any difference in reinfection-free survival between polymicrobial and monomicrobial FCPHIs in this series (14 vs. 51)^1^. The reinfection-free success rates were 100% for polymicrobial PHIs and 94% (±0.03) for monomicrobial PHIs at a 2-year follow-up (log-rank, *p* = 0.347).

Few data in the literature are available on polymicrobial prosthetic joint infections. They are limited, divergent, and mostly concern prosthetic joint infections treated with two-stage exchange arthroplasty (42–45). Data on polymicrobial PHIs with fistula are rare and do not bring details to compare with our outcomes (7, 20).

Another feature of our study is the administration of preoperative antibiotic therapy to select patients (44%). This procedure was only used if the bacteriological results of the preoperative joint aspiration culture were consistent. Preoperative antibiotic therapy was initially used to avoid post-operative severe sepsis or septic shock. It also decreased local inflammation and facilitated the quality of surgical excision. To note, antibiotic treatment in PHI management is recommended in recent Spanish guidelines in patients undergoing one-stage exchange arthroplasty, 3–5 days before surgery if the etiological diagnosis has already been made, especially if it is caused by *S. aureus* and gram-negative bacteria (46). Nineteen out of 29 patients (66%) of this series underwent 1- to 5-day preoperative antimicrobial therapy and 10 (34%) more than 5 days.

When used, cement was never antibiotic-loaded in our practice, and prostheses were mostly cementless. Overall, the literature still lacks an appropriately sized randomized clinical trial to better support the use of antibiotic-loaded cement, which still remains a matter of debate (47–49).

Optimization of microbiological diagnosis and medical–surgical treatment (one-stage arthroplasty and extended IV and oral post-operative antibiotics) can account for the success of the one-stage exchange arthroplasty, including in FCPHIs.

The limitations of our study are the small size of the series, as well as its observational, monocentric, and non-comparative type. However, there are no randomized controlled studies assessing one-stage vs. two-stage surgery in the treatment of PHIs, either with or without fistula.

## Conclusion

One-stage exchange arthroplasty strategy for FCPHIs shows a good success rate similar to that of non-fistulizing PHIs. Systematic preoperative microbiological documentation with joint aspiration and, in some specific cases, the use of preoperative antibiotic therapy are among the optimizations accounting for the success of this strategy. In light of our results, we believe that the presence of a fistula is not, in itself, a contraindication to performing a one-stage exchange arthroplasty for PHIs.

## Data Availability Statement

The datasets for this article are not publicly available because the local regulatory requires to keep confidential subject's data. Hence only researchers working on this article have access to data but not the general public. Requests to access the datasets should be directed to ykerroumi@hopital-dcss.org.

## Ethics Statement

The studies involving human participants were reviewed and approved by Comité de protection des personnes Ile de France VI. The patients/participants provided their written informed consent to participate in this study. Written informed consent was obtained from the individuals for the publication of any potentially identifiable images or data included in this article.

## Author Contributions

SM: article writing. YK: data capture, literature bibliography, statistical analysis, and article writing. VM, LL, AM, and WG: article reviewing. VZ: article reviewing and correction. All authors contributed to the article and approved the submitted version.

## Conflict of Interest

The authors declare that the research was conducted in the absence of any commercial or financial relationships that could be construed as a potential conflict of interest. The handling editor declared a shared department or other, though no other collaboration, with one of the authors, SM, at the time of review.

## Footnotes

^1^Preoperative and intraoperative specimens culture was sterile for one patient.

## Abbreviations

PHI: periprosthetic hip infection
FCPHI: fistulizing chronic prosthetic hip infection
Sd: standard deviation
CRP: C-Reactive Protein
ASA score: American Society of Anesthesiologist score.
